# Macropinosomes are Key Players in Early *Shigella* Invasion and Vacuolar Escape in Epithelial Cells

**DOI:** 10.1371/journal.ppat.1005602

**Published:** 2016-05-16

**Authors:** Allon Weiner, Nora Mellouk, Noelia Lopez-Montero, Yuen-Yan Chang, Célia Souque, Christine Schmitt, Jost Enninga

**Affiliations:** 1 Institut Pasteur, Dynamics of Host-Pathogen interactions Unit, Paris, France; 2 Institut Pasteur, Ultrapole, Paris, France; Purdue University, UNITED STATES

## Abstract

Intracellular pathogens include all viruses, many bacteria and parasites capable of invading and surviving within host cells. Key to survival is the subversion of host cell pathways by the pathogen for the purpose of propagation and evading the immune system. The intracellular bacterium *Shigella flexneri*, the causative agent of bacillary dysentery, invades host cells in a vacuole that is subsequently ruptured to allow growth of the pathogen within the host cytoplasm. *S*. *flexneri* invasion has been classically described as a macropinocytosis-like process, however the underlying details and the role of macropinosomes in the intracellular bacterial lifestyle have remained elusive. We applied dynamic imaging and advanced large volume correlative light electron microscopy (CLEM) to study the highly transient events of *S*. *flexneri’s* early invasion into host epithelial cells and elucidate some of its fundamental features. First, we demonstrate a clear distinction between two compartments formed during the first step of invasion: the bacterial containing vacuole and surrounding macropinosomes, often considered identical. Next, we report a functional link between macropinosomes and the process of vacuolar rupture, demonstrating that rupture timing is dependent on the availability of macropinosomes as well as the activity of the small GTPase Rab11 recruited directly to macropinosomes. We go on to reveal that the bacterial containing vacuole and macropinosomes come into direct contact at the onset of vacuolar rupture. Finally, we demonstrate that *S*. *flexneri* does not subvert pre-existing host endocytic vesicles during the invasion steps leading to vacuolar rupture, and propose that macropinosomes are the major compartment involved in these events. These results provide the basis for a new model of the early steps of *S*. *flexneri* epithelial cell invasion, establishing a different view of the enigmatic process of cytoplasmic access by invasive bacterial pathogens.

## Author Summary

*Shigella flexneri* is an intracellular bacterial pathogen and the causative agent of bacillary dysentery. It possesses the ability to invade and propagate within human cells by injecting bacterial effector proteins directly into host cells. Shortly after entry within a vacuole, *S*. *flexneri* induces vacuolar rupture and escapes into the host cytosol via an unknown mechanism. Using large volume correlative light electron microscopy (CLEM) and dynamic microscopy we studied discrete and highly transient steps of *S*. *flexneri* early invasion in detail. We provide the first 3D high resolution view of the *S*. *flexneri* invasion site and of vacuolar rupture itself. We find that vesicles formed at the invasion site due to injected bacterial effectors, termed macropinosomes, are functionally involved in vacuolar rupture and come into direct contact with the bacterial containing vacuole during this process. This unique and surprising pathogenic strategy stands in stark contrast to other invasive pathogens that induce direct lysis of their surrounding vacuole via the action of destabilizing bacterial proteins.

## Introduction

The lifestyle of intracellular bacterial pathogens is generally divided into the following steps: contact and entry into the host cell, residence within a vacuole, escape into the cytosol or establishment of a membrane encased niche, and cell-to-cell spreading [1]. Some intracellular bacterial pathogens, called invasive bacteria, such as *Yersinia pseudotuberculosis*, *Listeria monocytogenes*, *Salmonella enterica* and *Shigella flexneri*, are able to induce their own entry into nonphagocytic host cells [2]. Invasive bacteria are thought to subvert various host endocytic pathways during their life cycle, allowing the establishment of their specific intracellular niche and evasion of host immunity [3],[4].

*S*. *flexneri* is a medically important pathogen [5] that uses a type III secretion system (T3SS) to translocate bacterial proteins called effectors into the host cell [6],[7]. Induction of macropinocytosis has been proposed as the invasion strategy for *S*. *flexneri* entry into non-phagocytic epithelial cells [2]. In short, effectors released upon cell contact induce major rearrangements of the host cell cytoskeleton, mainly polymerization of actin filaments to form bundles supporting membrane projections termed “ruffles” [8],[9],[10],[11]. This leads to the formation of large membrane protrusions, which form a pocket enclosing the bacteria and facilitating entry [12]. Such ruffles appear similar to those described for macropinocytosis, a classical non-selective cellular uptake mechanism of molecules into large, irregular shaped vesicles, termed macropinosomes, formed by the collapse and fusion of ruffles with the plasma membrane [13]. In this classic model [2], entry and macropinosome formation represent a single process. However, evidence exists that bacterial entry and membrane ruffling are associated with different bacterial effectors and host responses during *S*. *flexneri* invasion: for example, host vinculin is recruited specifically to entering bacteria [14], Rho-GTPase isoforms are recruited differentially to either entering bacteria or membrane ruffles [8], and the bacterial effector IpgD was shown to regulate ruffling morphology and is not required for bacterial entry [15],[16]. Entry has been proposed to occur initially via effector mediated contact of *S*. *flexneri* [17] to cholesterol rich lipid raft membrane domains [18] and to be mediated by specific receptors [19],[20] suggesting entry is akin to receptor mediated phagocytosis. In the case of *Salmonella enterica*, an invasive,T3SS-employing pathogen which shares many common aspects with *S*. *flexneri* entry into host cells, it was hypothesized that *Salmonella* containing vacuole and macropinosomes may be distinct, as they are sorted into different intracellular routes [21]. Thus, it appears that the classic model for *S*. *flexneri* invasion may be too simplistic, and a revised model could include two parallel processes: (i) bacterial entry and (ii) membrane ruffling, whose precise biological role in invasion has not been studied in detail.

Upon entry, *S*. *flexneri* reside within bacteria containing vacuoles (BCVs), followed by BCV rupture and escape into the cytosol within 10 minutes, a step crucial for the intracellular growth of *S*. *flexneri* [22]. Initially, it was thought that bacterial effectors, such as the translocator proteins IpaB and IpaC, induce *S*. *flexneri* vacuolar rupture [23]. For instance, IpaB was shown *in vitro* to oligomerize and insert into the plasma membrane of target cells, forming cation selective ion channels involved in vacuolar rupture [24]. In contrast, we recently showed that *S*. *flexneri* subverts host cell pathways for BCV rupture [25]. A siRNA screen aimed at identifying host proteins involved in vacuolar rupture yielded multiple hits related to membrane trafficking, including EEA1, SNX1, VAMP2, and the small Rab GTPases Rab4, Rab5 and Rab11. Rab5 and Rab11 were recruited to the invasion site, with Rab11 localized to a large area surrounding invading bacteria, but not to the forming BCV. Rab11 knockdown caused a strong delay in vacuolar rupture timing, providing a functional link between membrane trafficking at the invasion site and vacuolar rupture. Additionally, the bacterial effector IpgD, a PI(4,5)P_2_ phosphatase, was shown to regulate Rab11 recruitment and to be required for efficient vacuolar rupture [25]. How this subversion of host cell pathways by *S*. *flexneri* relates to bacterial entry and vacuolar rupture has remained unclear.

In this work, we applied dynamic imaging and advanced large volume correlative light electron microscopy (CLEM) in the form of focused ion beam/ scanning electron tomography (C-FIB/SET) to reveal key features of the pathogenic strategy employed by *S*. *flexneri*. We analyze the architecture of the *S*. *flexneri* invasion site in detail and reveal that it contains two distinct compartments, the BCV and macropinosomes. We demonstrate that Rab11 is recruited directly to macropinosomes and its activity is required for efficient vacuolar rupture. Finally, we reveal that the BCV and macropinosomes come into direct contact at the onset of vacuolar rupture. Our results represent a major step forward in understanding the mechanism of *S*. *flexneri* escape to the cytosol, and provide the basis for an updated model of *S*. *flexneri* invasion into epithelial cells.

## Results

### *S*. *flexneri* is surrounded by macropinosome-like vesicles at the invasion site but does not reside within them

**Fig 1:**
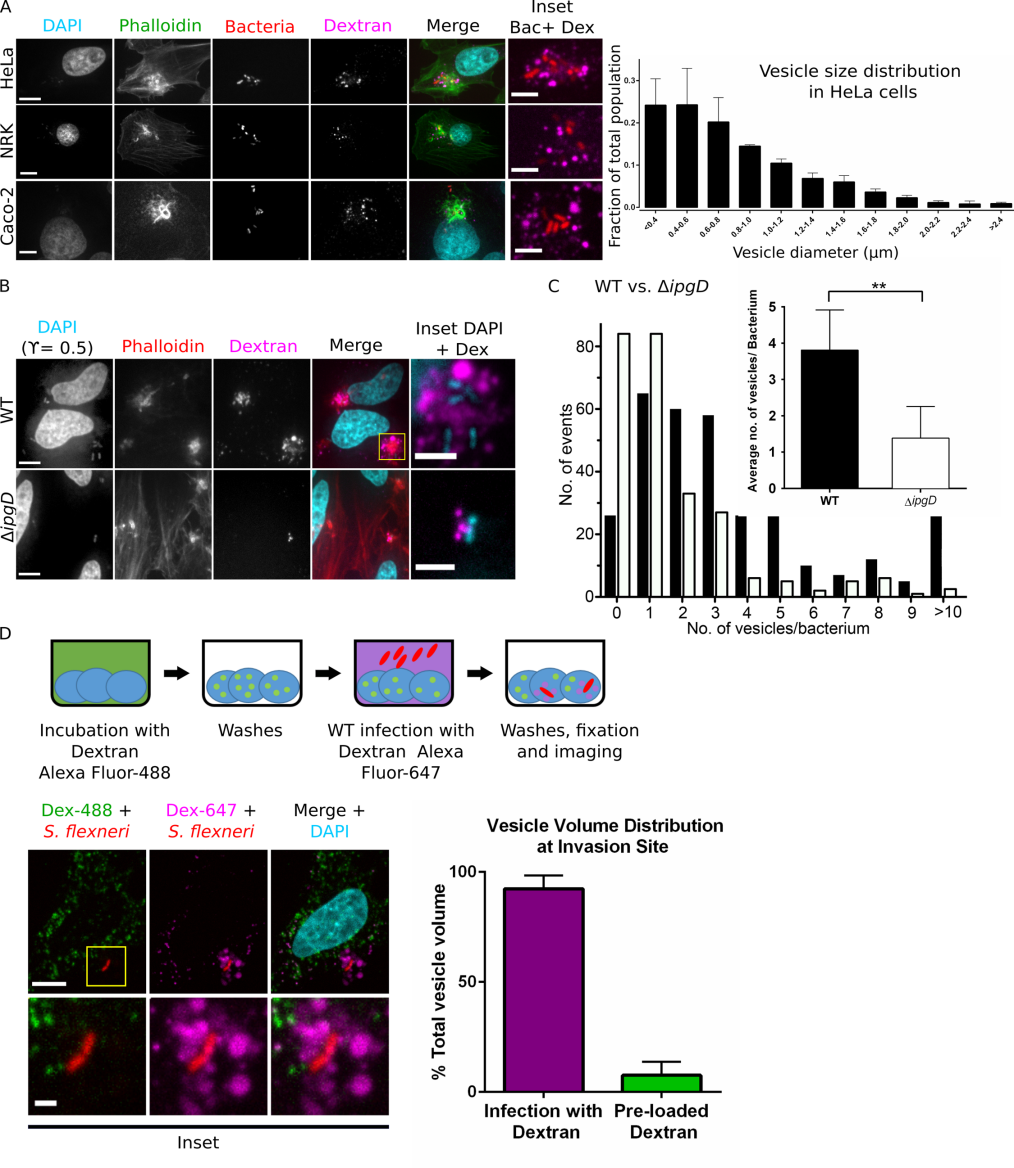
*S*. *flexneri* regulates macropinosome formation at the invasion site. (A) Confocal microscopy of the invasion site of WT fluorescent bacteria at 30 min infection in the presence of dextran Alexa Fluor-647. Hela, NRK and Caco-2 cells are compared. Actin rich invasion site ruffles are labeled with phalloidin. Scale bars are 10μm and 5μm (inset). Distribution of vesicle diameters at Hela cell invasion sites is presented (n = 2300 vesicles in 3 independent experiments). (B) WT compared to Δ*ipgD* invasion at 30 min infection. Bacteria are labeled with DAPI (0.5 ϒ-corrected images presented). Scale bars are 10μm and 2μm (inset). (C) Comparison of the number of vesicles per bacterium in WT vs. Δ*ipgD* strains. Distribution of all invasion sites analyzed (WT n = 324 sites, Δ*ipgD* n = 256 sites in six independent experiments) and averages are presented. (D) Sequential labeling experiment. Cells were incubated with dextran Alexa Fluor-488 before infection (green) then washed and infected in the presence of dextran Alexa Fluor-647 (magenta), washed, fixed and imaged. 92% of vesicle volume at the invasion site around bacteria (red) is occupied by vesicles formed during infection. (n = 476 vesicles in 34 invasion sites). Scale bars are 10μm and 2μm (inset). Z projections are presented in all panels.

Macropinosomes were previously observed at the invasion site of *Salmonella enterica* in epithelial cells using phase contrast microscopy and fluorescent dextran added to the infection media, acting as a fluid phase marker [21]. We set out to examine macropinosome formation at the *S*. *flexneri* invasion site by measuring internalization of fixable dextran conjugated to Alexa Fluor-647 (Fig 1A). HeLa, Caco-2 and NRK cells were infected for 30 minutes with wild-type dsRed expressing bacteria, added together with the fluorescent conjugate. This was followed by washes to remove non-internalized dextran, fixation and staining for actin to visualize the site of invasion, and DNA to visualize cell nuclei. Multiple dextran containing vesicles were observed surrounding *S*. *flexneri* at the invasion site to a similar extent in all host cell types used. The size distribution of the vesicles was quantified in HeLa cells using automated software. Vesicles are heterogeneous in size, with the majority of vesicles having a diameter less than 1μm and a fraction of larger vesicles observed. As *S*. *flexneri* is generally thought to enter cells within a pocket formed by large membrane protrusions [12], we expected the BCV to also contain detectable fluid phase marker. Surprisingly, bacteria were never observed within dextran positive vesicles, implying *S*. *flexneri* containing vacuoles exclude most extracellular fluid. The observed vesicle size heterogeneity and uptake of extracellular fluid are reminiscent of features classically associated with macropinosomes [26], suggesting *S*. *flexneri* is surrounded by macropinosome-like vesicles (here on referred to simply as ‘macropinosomes’, see discussion) at the invasion site but does not reside within them.

### The bacterial effector IpgD regulates macropinosome formation

As the bacterial effector IpgD has been previously implicated in regulation of ruffling morphology during *S*. *flexneri* invasion [15],[27], we examined its role in macropinosome formation (Fig 1B and 1C). Confocal microscopy of HeLa cells infected by wild-type and Δ*ipgD* strains in the presence of dextran Alexa Fluor-647 for 30 minutes revealed a reduction in the number of macropinosomes surrounding bacteria when using the mutant strain (Fig 1B). Automated quantification of the number of macropinosomes formed per bacterium revealed that macropinosome formation was reduced by around 60% when using the IpgD mutant (Fig 1C). Infection with Δ*ipgD*/IpgD strain showed partial complementation of vesicle formation, in accordance with previously reported results [25] (S1 Fig). We conclude that macropinosome formation at the *S*. *flexneri* invasion site is regulated by the bacterial effector IpgD.

### Newly formed macropinosomes are the major compartment at the *S*. *flexneri* invasion site

Intracellular bacterial pathogens are normally thought to subvert pre-existing host endocytic vesicles during invasion (see introduction), however our study indicated that nascent macropinosomes appear to be a major endomembrane component at the invasion site of *S*. *flexneri*. We examined the relative contribution of pre-existing endocytic vesicles to the endomembrane content at the invasion site (Fig 1D). To this end, we performed sequential labeling experiments, pre-loading cells with dextran Alexa Fluor-488 for two to three hours prior to infection to allow uptake by the host endocytic compartment. This was followed by extensive washes and infection in the presence of dextran Alexa Fluor-647. Extensive labeling of punctate dextran Alexa Fluor-488 endomembrane structures was observed throughout the cells. Strikingly, we could not detect accumulation of dextran Alexa Fluor-488 containing vesicles around invading bacteria, while dextran Alexa Fluor-647 vesicles were highly enriched around bacteria. Furthermore, we did not detect any co-localization of the two differently colored dextrans around the entering bacteria at the measured time-point. Quantitative image analysis revealed 92% of total vesicle volume (i.e. combined volume of vesicles labeled by either dextran Alexa Fluor-488 or dextran Alexa Fluor-647) at the invasion site is occupied by dextran Alexa Fluor-647 containing vesicles. This indicated that the local environment around invading *S*. *flexneri* is occupied by newly formed macropinosomes, without significant recruitment of pre-existing host endocytic vesicles, arguing against active subversion of pre-existing host endocytic pathways by *S*. *flexneri* during invasion.

### C-FIB/SET provides detailed 3D structural information of discreet and transient stages of *S*. *flexneri* invasion

**Fig 2:**
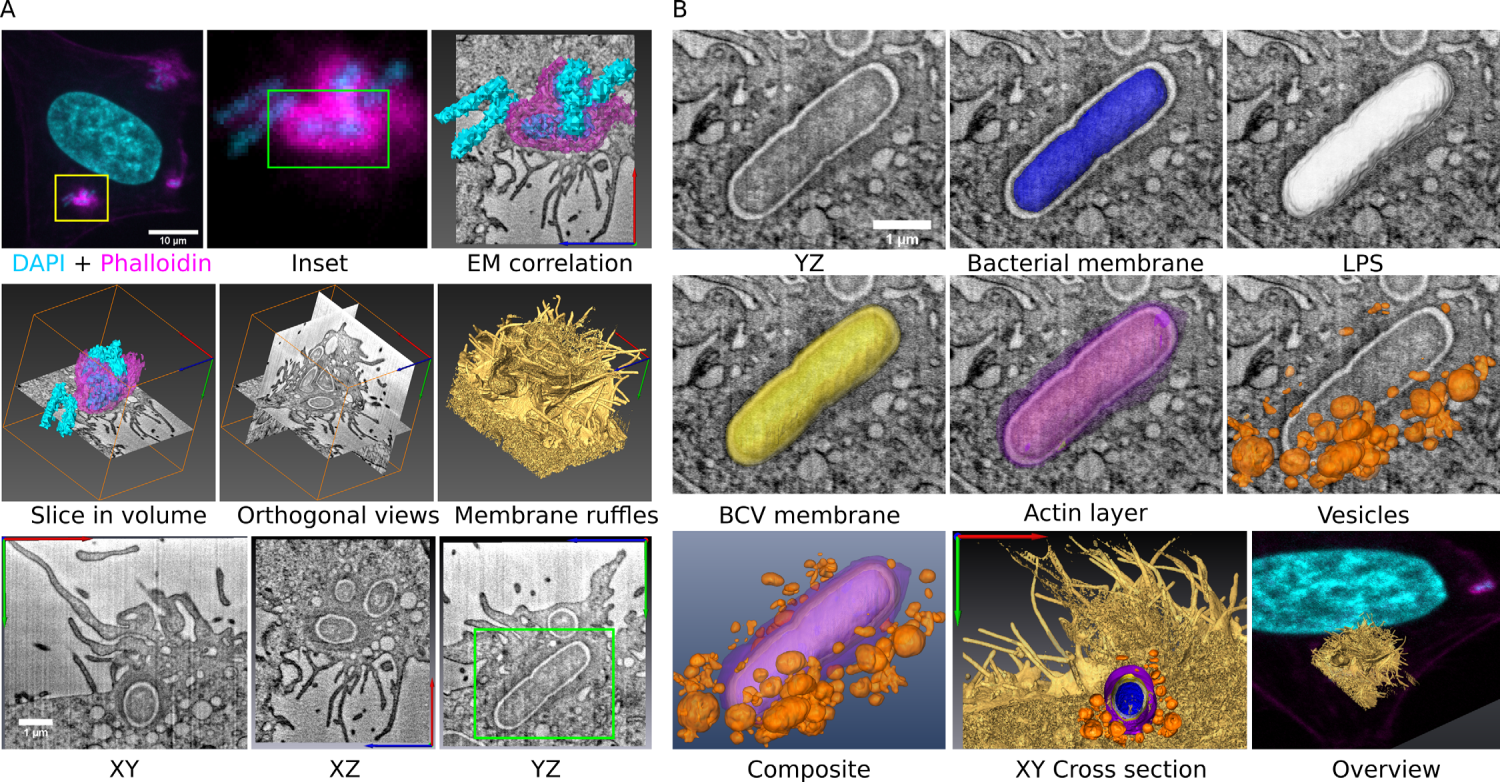
C-FIB/SET reveals the BCV is a tight compartment structurally distinct from surrounding vesicles. (A) Confocal microscopy of an early stage invasion site, labeled for actin (magenta) and DNA (cyan). Actin enrichment is observed around a bacterium at the site of interest (Inset, green box). This site is correlated with the corresponding 3D FIB/SET data allowing ultrastructural visualization of the entire invasion site, presented in three orthogonal views. Surface membrane ruffling (gold) and inner cell structures are observed in detail. The same bacterium is identified (panel YZ, green) in division at the center of the volume. See also S1 Movie. (B) Typical structural components of the *S*. *flexneri* BCV. Bacterial cytosol and membrane (blue); LPS layer (white); BCV membrane (yellow); and an actin layer (magenta), observed only prior to vacuolar rupture. The BCV is surrounded by vesicles (orange). Additional views place BCV in cellular context. See also S2 Movie. Overall 15 FIB/SET data sets of invasion sites were acquired.

Since fine structural details of the *S*. *flexneri* invasion site are obscured by the resolution limit of standard light microscopy, we applied CLEM in the form of correlative large volume focused ion beam/scanning electron tomography (C-FIB/SET) to examine the *S*. *flexneri* invasion site in detail [28] (Fig 2). This emerging technique combines 3D fluorescent and large volume electron tomography into a single correlated data set, providing three features ideal for investigation of *S*. *flexneri* invasion: First, discreet and highly transient (in the order of minutes) stages of invasion can be targeted using fluorescent microscopy with stage-specific fluorescent markers prior to tomography acquisition. Thus, precise access to early invasion (before BCV rupture) and to the vacuolar rupture event itself can be gained. Secondly, the large cellular volumes (in the order of 1000 μm^3^) acquired using this technique allow visualization of entire invasion sites, accounting for multiple bacteria, vesicles and other cellular structures present at the site. The BCV’s and other vesicles’ membranal integrity and their connectivity to other compartments can be examined in 3D from all axes, providing structural information not easily accessible by classic serial sectioning and tomography approaches [29]. Finally, the fluorescent labeling correlated in 3D to the tomography data (with bacteria observed by light and electron microscopy acting as alignment fiducials) provides molecular specificity to features observed within the tomography volume, albeit within the light microscopy diffraction limit (see materials and methods). Overall, 15 C-FIB/SET data sets of invasion sites were acquired for this study. As fluorescence microscopy of the *S*. *flexneri* invasion site revealed that BCVs exclude detectable fluorescent dextran and are surrounded by dextran containing vesicles in all our experiments, we first set out to examine the architecture of the early invasion site in detail using C-FIB/SET. A detailed description of the correlative workflow for the data set analyzed in Fig 2 is presented (Fig 2A, S1 Movie): First, a *S*. *flexneri* invasion site was imaged by confocal microscopy. As a tight actin enrichment around bacteria was previously shown to appear exclusively prior to vacuolar rupture [30], a site containing bacteria with this feature (as indicated by phalloidin staining) was chosen, thereby providing a clear indication of early invasion (Fig 2A, inset). After sample processing for electron microscopy, a FIB/SET data set was acquired at the exact same location. The two data sets were then combined into a single correlated volume, providing a full 3D view of the entire *S*. *flexneri* invasion site from all axes. Membrane ruffles are observed at the cell surface while BCVs and smaller vesicles are seen in the cytosol.

### The BCV is a tight compartment distinct from surrounding macropinosomes

We examined the structure of the BCV in early invasion in detail and delineated the typical BCV structural motifs (Fig 2B, S2 Movie). The data in Fig 2B is representative of all datasets acquired during early invasion in our study. We found that the bacterial cytosol and membrane are surrounded by a layer of low electron density. Based on annotation from previous ultrastructural studies we associate this layer with bacterial lipopolysaccharides (LPS) (e.g. [31]). Furthermore, this layer is also present in rupturing BCVs missing parts of their BCV membrane (see below), indicating it does not represent the vacuole lumen. These bacterial structures are tightly enclosed by the vacuole membrane. An additional electron dense layer observed only around bacteria surrounded by phalloidin stain (as identified by fluorescence microscopy) is consequently identified as actin. Finally, we observe multiple small vesicles surrounding the BCV, but not contacting it nor each other. In all data sets observed, BCVs contained a single bacterium (often in division), were structurally uniform and tightly surrounding the bacterium inside. We were not able to detect a luminal BCV space around the bacteria in any of our data sets. Overall, our high resolution correlated view of the early *S*. *flexneri* invasion site reveals the BCV is a tight, uniform compartment, structurally distinct from the surrounding heterogeneous macropinosomes. We conclude that the BCV and macropinosomes present at the early invasion site represent two distinct compartments.

### C-FIB/SET confirms the presence of newly formed macropinosomes at the invasion site

Our two color dextran labeling experiments (see Fig 1D), indicated that the local environment around invading *S*. *flexneri* is occupied by macropinosomes formed *in situ* during invasion without significant recruitment of pre-existing host endocytic vesicles. We applied C-FIB/SET to correlate the total vesicle population at the invasion site with dextran Alexa Fluor-647 labeling (added during infection) (S2 Fig). FIB/SET analysis reveals the entire vesicle population around invading *S*. *flexneri* irrespective of the fluorescent probe, while the dextran Alexa Fluor-647 label provides an indication of the volume occupied by vesicles formed during invasion. In the two datasets presented (S2A and S2B Fig), 95% and 99% of vesicles found at the invasion site reside within the fluorescent dextran label (S2C Fig). In data set B, five of the largest vesicles, found near the surface of the cell, lie outside of the dextran labeling, most likely representing late forming macropinosomes formed after removal of dextran in the washing phase. This result confirms that newly formed macropinosomes are the major compartment at the *S*. *flexneri* invasion site, in agreement with our two color dextran labeling experiments (Fig 1D).

### Vacuolar rupture efficiency is correlated to macropinosome availability

**Fig 3:**
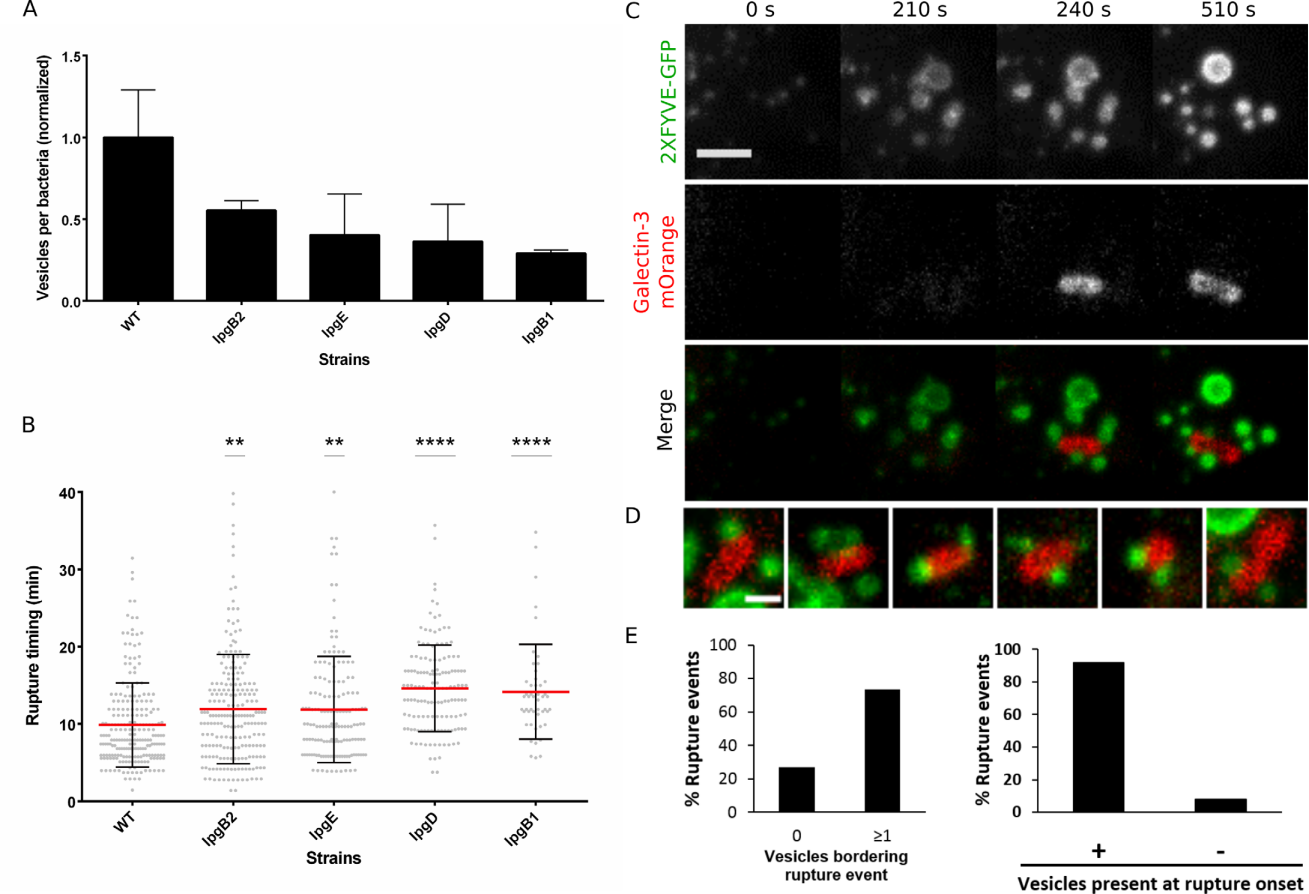
Macropinosome availability is correlated with the efficiency of vacuolar rupture and macropinosomes border the BCV at the onset of vacuolar rupture. (A) Effector mutant library macropinosome formation screen. Number of macropinosome per bacteria at the invasion site was quantified for infection with WT and various effector mutant strains (n≥70 invasion sites per strain from 2 independent experiments). (B) Effector mutant library vacuolar rupture timing screen (n≥50 invasion events per strain, from 3 independent experiments. Significance of mutant vs. WT was determined using one way ANOVA.). Each data point represents a single rupture event recorded during live imaging. (C) Confocal time-lapse microscopy of cells transfected with the PI3P macropinosome marker 2XFYVE-GFP and vacuolar rupture marker galectin-3-mOrange, and infected with WT bacteria. Typical events are presented: onset of membrane ruffling (0 s), formation of macropinosomes (210 s), vacuolar rupture in close proximity to macropinosomes (240 s), ongoing macropinosome presence and progression of rupture (510 s). Images were taken every 30 s, z-projections are presented, scale bar is 5μm. See also S3 Movie. (D) Gallery of different rupture events where macropinosomes (labeled by 2XFYVE-GFP) border rupturing BCVs (labeled by galectin-3-mOrange). Scale bar is 2 μm. (E) Frequency of macropinosomes bordering (within 1 μm) the rupturing BCV from total rupture events observed (left). In 73% of vacuolar rupture events analyzed macropinosomes were found bordering the BCV (n = 30 rupture events in 3 independent experiments). Frequency of vesicles present at rupture onset based on high temporal resolution imaging (right). Macropinosome were found at least one frame before rupture in 92% of rupture events containing bordering macropinosomes (n = 36 rupture events in eight independent experiments).

We have recently shown that the bacterial effector IpgD is required for efficient vacuolar rupture [25]. As IpgD is also a regulator of macropinosome formation (see Fig 1B and 1C), we hypothesized these two processes may be functionally related. In order to test this hypothesis, we performed two bacterial effector mutant library screens aimed at identifying the relationship between the number of macropinosomes at the invasion site and vacuolar rupture timing (Fig 3A and 3B, selected hits are presented. For screen details see materials and methods. Full screen results are provided in S3A Fig). First, the library was screened for the number of macropinosomes formed at the invasion site using quantitative image analysis of fluorescent dextran containing vesicles (Fig 3A). Various effectors alongside IpgD were found to affect macropinosome formation, including IpgB1, IpgE (a chaperone for IpgD [15]) and IpgB2. Secondly, the library was screened for vacuolar rupture timing using dynamic microscopy (Fig 3B). We found an inverse correlation between the number of macropinosomes formed at the invasion site and the delay in vacuolar rupture timing, i.e. the fewer macropinosomes found at the invasion site, the longer rupture is delayed. Of particular interest is the effector IpgB1, the strongest hit in both screens. IpgB1 was shown to be involved in the early stages of *S*. *flexneri* invasion, is associated with membrane ruffles, and can induce membrane ruffling via stimulation of Rac1 and Cdc42 activities. Like IpgD, it has no known function in disrupting membrane integrity or pore formation to our knowledge [32]. Thus, our mutant screens results revealed that vacuolar rupture efficiency is correlated to macropinosome availability. We also studied the effect of drugs known to inhibit macropinocytosis and actin dynamics on macropinosome formation and vacuolar rupture timing. Amiloride, a Na(+)/H(+) exchange inhibitor known to cause indirect inhibition of macropinocytosis [33] did not inhibit macropinosome formation and entry at low doses and was cytotoxic at higher doses. As we have previously identified via high-content siRNA screening that Arp2/3 subunits are among host proteins involved in *S*. *flexneri* uptake and vacuolar rupture [25] we tested CK-666, an Arp2/3 complex inhibitor that does not stimulate dissociation of preformed actin branches in vitro [34]. CK-666 inhibited macropinosome formation without inhibiting bacterial entry, and significantly delayed vacuolar rupture timing (S3B Fig). The selective inhibition of macropinosome formation by CK-666 may be due to the drug having a stronger effect on ruffle formation, a process requiring massive actin re-arrangement, as opposed to the more small scale actin re-arrangement required for bacterial uptake. Overall, our results demonstrate that vacuolar rupture timing is dependent on the availability of macropinosomes, indicating that membrane ruffling and macropinosome formation may be functionally linked to vacuolar rupture.

### Time lapse microscopy reveals macropinosomes border the BCVs at the onset of vacuolar rupture

We therefore set out to examine the cell biology underlying the relation between macropinosomes and vacuolar rupture in detail using dynamic microscopy, functional assays and structural analysis. First, we examined the spatial and temporal proximity of macropinosomes to the rupturing BCV. We performed live microscopy of *S*. *flexneri* invasion in cells transfected with a marker of phosphatidylinositol 3-phosphate (PI3P), 2XFYVE-GFP, used for labeling macropinosomes, and galectin-3-mOrange used for labeling vacuolar rupture. PI3P has been previously reported to be associated with early macropinosome maturation, in particular with the cup closure step [35]. We found that 2XFYVE-GFP was partially co-localized with dextran positive vesicles at the invasion site, this partial co-localization likely due to the transient nature of its recruitment to forming macropinosomes [35] (S3C Fig). Galectin-3-mOrange is commonly used as a vacuolar rupture marker as it specifically labels the BCV only after loss of vacuole integrity [36]. 3D multi-channel images were acquired every 30 seconds (Fig 3C and 3D, S3 Movie) and the resulting movies were quantified by counting the number of events where macropinosomes were found bordering (within 1μm) the rupturing BCV, as indicated by the first appearance of a galectin-3-mOrange signal (Fig 3E left). In 73% of all vacuolar rupture events analyzed (n = 30 events in three independent experiments), macropinosomes were found bordering the BCV, with 50% of events containing more than one bordering vesicle. This number most likely underrepresents such events as not all macropinosomes are labeled with 2XFYVE-GFP. In order to assess whether macropinosome proximity to the BCV occurs upstream of rupture or is a product of rupture, we performed high temporal resolution imaging with multi-channel confocal stacks acquired every 5 seconds (S3D Fig, S4 Movie). This allowed clear detection of bordering macropinosomes at the onset of the galection-3-mOrange signal appearance and therefore at the onset of vacuolar rupture. Data quantification (Fig 3E, right) showed that in 92% of the subset of vacuolar rupture events containing bordering macropinosomes (n = 36 events in three independent experiments), bordering occurred at least one frame before the onset of rupture, indicating this proximity is present upstream of vacuolar rupture. In all movies acquired, rupturing BCV’s (i.e. labeled with galectin-3-mOrange) were never co-labeled with 2XFYVE-GFP, and 2XFYVE-GFP positive vesicles were never co-labeled with galection-3-mOrange, implying macropinosome proximity to the rupturing BCV does not result in membrane fusion or ruptured macropinosomes (see discussion).

### Host Rab-GTPases are recruited to macropinosomes

Rab-GTPases act as markers for macropinosome formation and maturation [13],[37] and have been previously shown to be recruited to the *S*. *flexneri* invasion site to various degrees [25]. We examined the recruitment of Rabs to the invasion site in the context of dextran labeled macropinosomes and invading bacteria (S4A Fig). Cells transfected with Rab4-GFP, Rab5-GFP, Rab7-GFP and Rab11-GFP were infected with wild-type fluorescent bacteria for 30 minutes in the presence of dextran Alexa Fluor-647. Rab4-GFP was not recruited to the invasion site, in agreement with previous studies [25], while other Rabs examined were recruited to macropinosomes at the invasion site to various degrees but not around invading bacteria. As Rab11’s association with macropinosomes has not been previously demonstrated in other systems to our knowledge, and its recruitment has been previously shown to be prevalent at the invasion site and is required for efficient vacuolar rupture [25], we decided to examine its association with macropinosomes in detail.

### Rab11 is directly and dynamically recruited to macropinosomes

**Fig 4:**
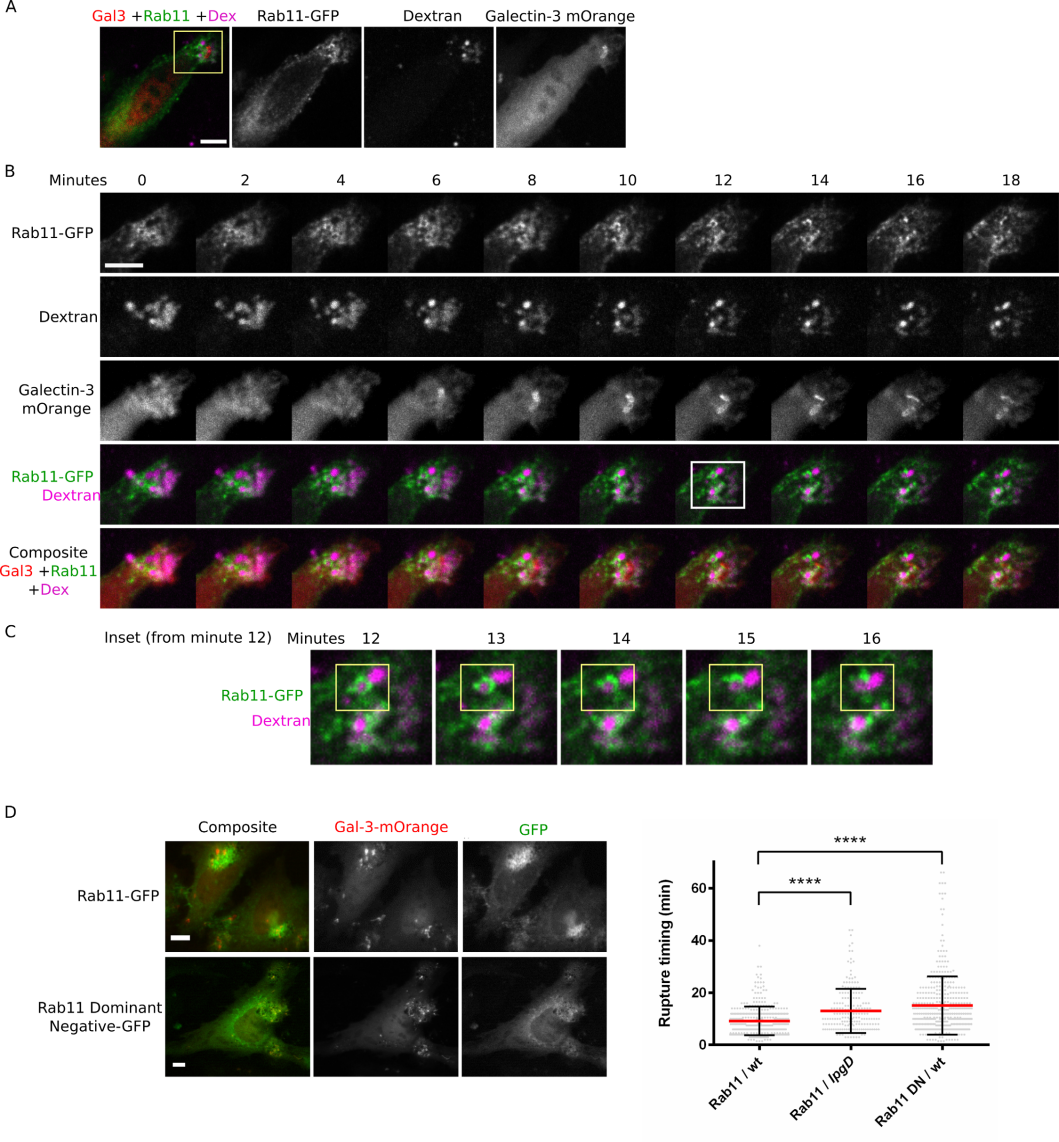
Rab11 is directly and dynamically recruited to macropinosomes and its activity is required for efficient vacuolar rupture. (A) Dextran pulse chase live imaging experiment. Overview of invasion site. (B) Time series of *S*. *flexneri* invasion. Inset from A is presented. Minute 0 represents the time when dextran was washed out and imaging was initiated. The onset of vacuolar rupture is observed in frame “6”. (C) Inset from B showing Rab11 is dynamically and directly recruited to a dextran labeled macropinosome (highlighted in yellow). Max projection of three slices is presented. See also S5 Movie. (D) Rab11S25N-GFP GDP locked dominant negative (Rab11 DN) is not recruited to invasion site and causes a significant delay in vacuolar rupture timing. Cells were transfected with Rab11-GFP or Rab11 DN and galectin-3-mOrange followed by live imaging during *S*. *flexneri* invasion. Two representative single slices from movies showing the localization of Rab11 and Rab11 DN are presented (left). Comparison of vacuolar rupture timing (right). Wild-type infections of Rab11-GFP transfected (Rab11/wt) and Rab11 DN (Rab11 DN/wt) cells are presented. Δ*ipgD* infection of Rab11-GFP transfected cells (Rab11/IpgD) is used as a control. Data obtained from three independent experiments with 946 overall rupture events measured. See also S6 Movie, S7 Movie. Scale bars in all panels are 10 μm.

While Rab11 is classically associated with recycling endosomes [38],[39], our results suggested that the major compartment at the invasion site is composed of macropinosomes and that host endosomes are not recruited during invasion (Fig 1D, S2 Fig). Furthermore, our fixed invasion experiments (S4A Fig) revealed some macropinosomes directly surrounded by Rab11-GFP. Taken together, these results suggest that during invasion Rab11 can associate directly with newly formed macropinosomes via a non-classical pathway that does not involve recycling endosomes. In order to test this hypothesis we performed dextran pulse chase experiments combined with live imaging of cells transfected with Rab11-GFP and galectin-3-mOrange (Fig 4A–4C, S5 Movie). Cells were infected with wild type *S*. *flexneri* in the presence of dextran Alexa Fluor 647 for 22 minutes. At that point the cells were quickly washed to remove non-internalized dextran from the media and live imaging was initiated. Cells were imaged in 3D at 60s intervals. The time point for dextran washes (t = 0) was chosen so that invasion sites already containing internalized dextran but prior to vacuolar rupture (as indicated by galectin-3-mOrange) could be captured. Thus the relation between dextran containing macropinosomes and Rab11-GFP recruited to the invasion site could be examined during live imaging of early invasion. A representative experiment is presented. We found that Rab11 is directly and dynamically recruited to macropinosomes prior and during vacuolar rupture, conforming the non-classical association between macropinosomes and Rab11 at the *S*. *flexneri* invasion site. As expected, live imaging also revealed that rupture events occur in close proximity to macropinosomes (in agreement with Fig 3C–3E).

### Rab11 activity is required for efficient vacuolar rupture but not for macropinosome formation

Given the non-classical association between macropinosomes and Rab11 occurring during *S*. *flexneri* invasion, we examined the functional role of Rab11 in relation to macropinosome formation and vacuolar rupture. We employed a functionally impaired Rab11: Rab11S25N-GFP, a GDP-locked dominant negative (Rab11 DN) [40]. We found that it did not inhibit macropinosome formation or bacterial entry, and unlike Rab11-GFP, was not recruited to macropinosomes (Fig 4D left, S4B and S4C Fig). Next we examined the functional role of Rab11 in vacuolar rupture. We transfected cells with Rab11-GFP or Rab11 DN and galectin-3-mOrange and performed live imaging of *S*. *flexneri* invasion to determine vacuolar rupture timing (Fig 4D right, S6 Movie, S7 Movie showing representative experiments). We found that vacuolar rupture is significantly delayed when using the Rab11 DN. Our results indicate that while Rab11 is not required for macropinosome formation or bacterial entry, its activity is required for recruitment to macropinosomes and efficient vacuolar rupture. We hypothesize that Rab11’s role in vacuolar rupture is manifested through its well described function in vesicle trafficking regulation [39] acting uniquely on macropinosomes in the case of *S*. *flexneri* invasion (see discussion).

Overall our effector mutant screens, live imaging and functional studies reveal that macropinosomes at the invasion site are the target for direct recruitment of Rabs, they border the BCVs at the onset of vacuolar rupture, and their formation and trafficking are required for efficient vacuolar rupture. Together, these results provide for the first time evidence that vacuolar rupture involves two cellular compartments, the BCV and macropinosomes.

### Macropinosomes come into direct contact with the bacteria containing vacuole during vacuolar rupture

**Fig 5:**
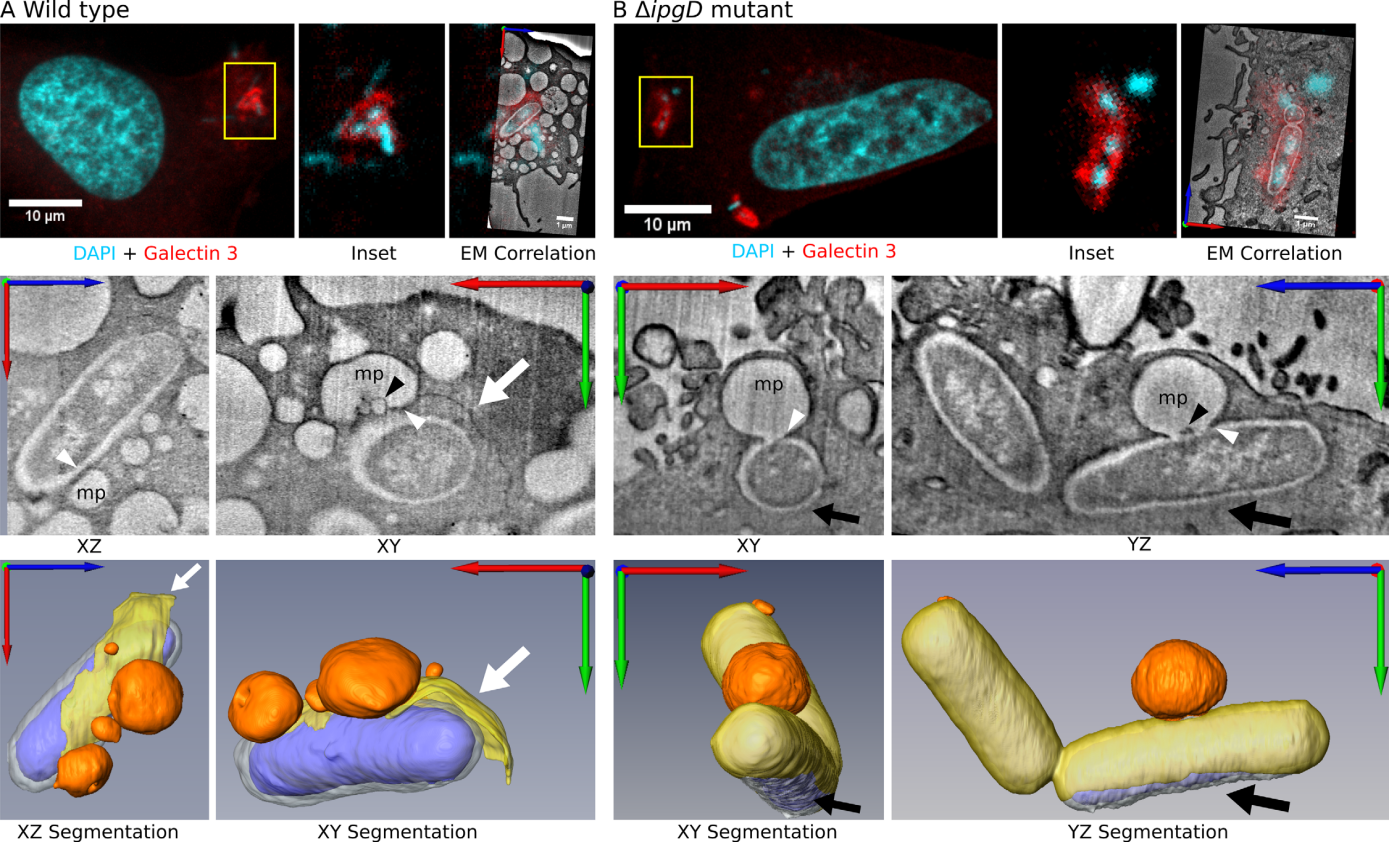
Macropinosomes come into direct contact with the bacteria containing vacuole during vacuolar rupture. Vacuolar rupture occurring during of WT and Δ*ipgD* infections was studied using C-FIB/SET. Confocal microscopy was used to identify bacteria (cyan) within rupturing BCVs labeled by galectin-3-mOrange (red). (A) 3D ultrastructural data of WT reveals several macropinosomes (mp, orange) in direct contact (white arrow heads) with the BCV membrane (yellow), surrounding the bacteria (blue -cytosol, white- LPS). A dissociated BCV membrane is observed (A, white arrow) as well as smaller intraluminal vesicles within the macropinosome (A, black arrowhead). See also S8 Movie. (B) The Δ*ipgD* strain exhibits less vesicles in contact with the BCV than the Δ*ipgD* mutant strain, yet contact morphology is maintained (white arrow heads). Rupture on the opposing end to the contact point is observed (black arrow). See also S9 Movie. Overall 15 FIB/SET data sets of invasion sites were acquired (11 WT, 4 Δ*ipgD)*. For each strain, a representative data set of a site containing a rupturing BCV is presented.

We hypothesized that macropinosomes are directly implicated in vacuolar rupture via physical contacts with the BCV. Such contacts are obscured when using fluorescence microscopy due to the limited resolution of light microscopy. We therefore applied C-FIB/SET to image the very short-lived and highly dynamic rupturing event in detail, acquiring correlative data sets of invasion sites containing BCVs labeled with galectin-3-mOrange to indicate rupture (Fig 5). C-FIB/SET is particularly suited for the unambiguous identification of compartmental contact points at the invasion site, as it provides information in all three axes and within a large cellular volume likely to contain multiple contacts occurring at various orientations. Strikingly, in cells infected with the wild-type strain (Fig 5A, S8 Movie) rupturing BCVs were found in direct contact with multiple surrounding vesicles. A partly dissociated BCV membrane emanating into the cytosol from the contact point between a macropinosome and a BCV was observed, with most of it still attached to the bacterium (Fig 5A, white arrows, yellow). Smaller intraluminal vesicles within the large macropinosome were commonly observed at the interface between the macropinosome and the BCV (Fig 5A, black arrowhead). As IpgD is required for efficient rupture [25], we examined its involvement in the formation of macropinosome-BCV contact points. Infection with a *Δipg*D strain revealed a reduction in the number of vesicles in contact with the BCV, yet macropinosome-BCV contact points morphologically identical to the wild-type were still always observed (Fig 5B, S9 Movie), indicating that IpgD regulates macropinosome availability but does not impact the formation of macropinosome-BCV contacts during rupture. In the rupture events presented here, most of the BCV membrane is still present (Fig 5B, yellow), and rupture is observed at the opposite side of the bacterium in relation to the contact points (Fig 5B, black arrows). We conclude that the BCV and surrounding macropinosomes come into direct contact during vacuolar rupture. Thus, our functional, dynamic and structural analysis (Figs 3, 4 and 5) suggest that macropinosomes are required for efficient vacuolar rupture and that macropinosome-BCV contacts are involved in this process.

## Discussion

**Fig 6:**
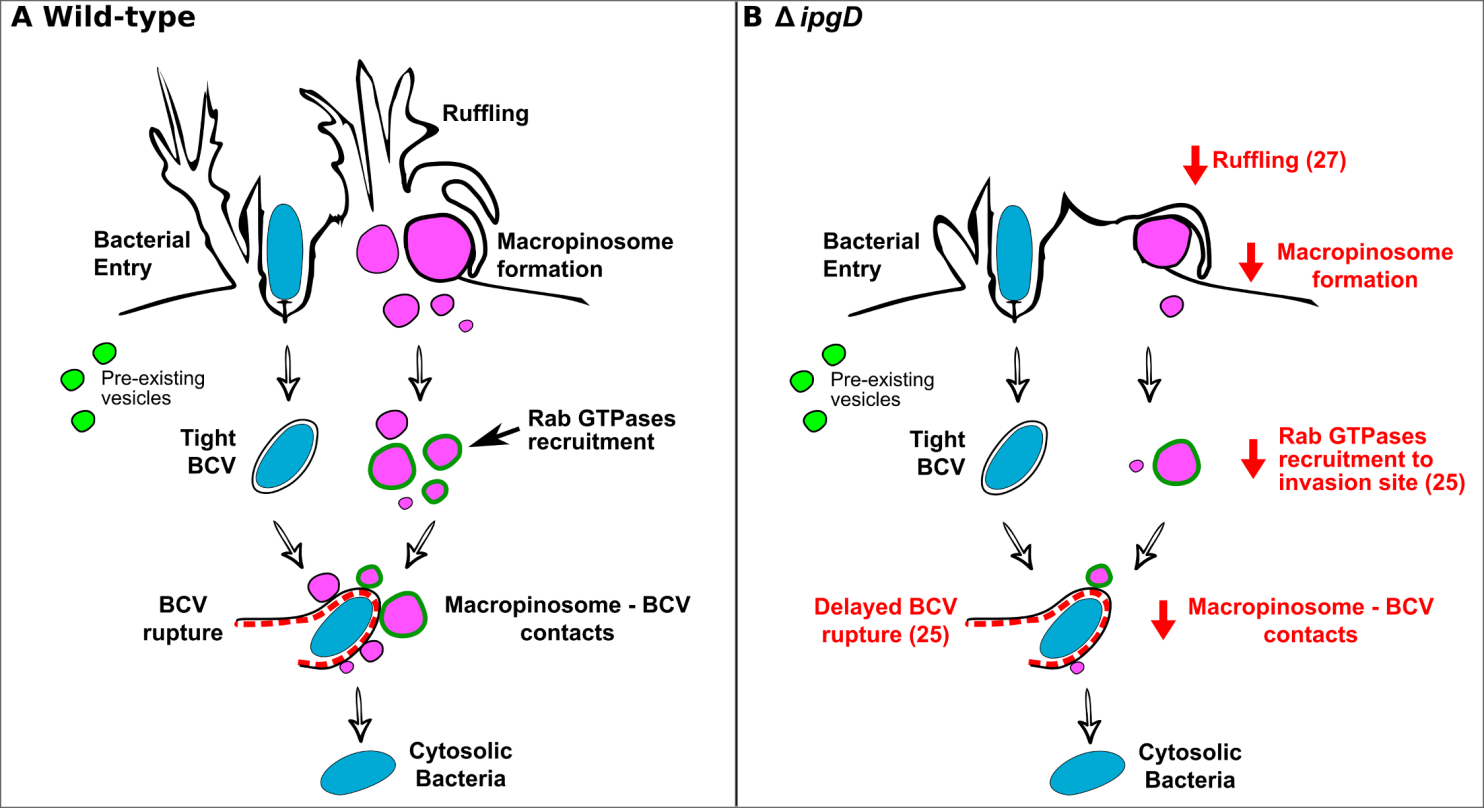
New model of *S*. *flexneri* early invasion into epithelial cells. Wild-type (A) is compared with Δ*ipgD* (B). Altered steps are indicated in red with references to relevant work. *S*. *flexneri* (blue) induces membrane ruffling resulting in macropinosome formation (pink) in conjunction with its entry into a tight BCV. Rab GTPases (dark green) are then recruited to macropinosomes. Finally macropinosomes and the BCV come into direct contact at the onset of vacuolar rupture (dashed line) and bacteria escape to the cytosol. Pre-existing endocytic vesicles (light green) are not recruited to the invasion site. Δ*ipgD* reduces membrane ruffling, resulting in fewer macropinosomes that can then be targeted by Rab GTPases (observed as on overall reduction of Rab recruitment to the invasion site). As a result less macropinosome- BCV contacts are formed and vacuolar ruptured is delayed.

We report here that macropinosomes are a central compartment in *S*. *flexneri* invasion and that they are implicated in *S*. *flexneri* vacuolar rupture. Our results lay the ground for a new model of the early steps of *S*. *flexneri* invasion into epithelial cells (Fig 6A): Invasion begins with two distinct processes occurring in conjunction: bacterial entry into a tight BCV and the regulated formation of macropinosomes via membrane ruffling. Then, host Rab GTPases are recruited directly to nascent macropinosomes, followed by direct contact between macropinosomes and the BCV at the onset vacuolar rupture. Host endomembranes are not recruited to the invasion site, indicating invasion occurs without subversion of pre-existing host endocytic pathways or pre-existing endomembrane compartments. Macropinosomes are revealed to be key players in the process of vacuolar rupture, as indicated by functional experiments demonstrating that the efficiency of vacuolar rupture is dependent on macropinosome availability and Rab11 activity (acting in direct association with macropinosomes). This new model provides a conceptual framework that accommodates previous studies [8],[14],[15],[16],[25], and has predictive power in regard to the possible biological role of membrane ruffling and macropinosomes in other pathogenic systems [11],[21]. For example, various observations made regarding the bacterial effector IpgD are put into biological context with our work: While IpgD is not required for bacterial entry [15], it is known to promote ruffling [15], is required for macropinosome formation (Fig 1B and 1C), Rab11 recruitment to the invasion site and efficient vacuolar rupture [25],[27]. These activities are now placed in sequence, as ruffling, macropinosome formation, Rab GTPase recruitment to macropinosomes and vacuolar rupture represent a cascade of causally connected events occurring during *S*. *flexneri* invasion (Fig 6B).

Vesicles induced by *S*. *flexneri* share some similarities with classically described macropinosomes formed in non-pathogenic systems [13],[41]. Their formation in the context of membrane ruffling, size heterogeneity and uptake of fluid phase marker resemble that of macropinosomes (and micropinosomes, accounting for smaller vesicles formed at the invasion site). However, their formation is induced via the action of bacterial effectors that modulate other cellular pathways as well, and thus may affect vesicle biochemistry. Indeed, our results demonstrate that *S*. *flexneri* induced macropinosomes are associated with Rab11, a typical marker for the recycling endosomal pathway [38],[39], not known to label macropinosomes in other systems. Macropinosome trafficking in the absence of pathogens is cell type dependent, and while professional phagocytizing cells direct macropinosomes into the lysosomal pathway [42], in some non-professional phagocytizing cells, macropinosomes were reported to recycle to the plasma membrane, with little or no interaction with endosomal vesicles [41],[43],[44]. It may be that the macropinosome-BCV contacts are a product of “misdirected” trafficking towards the BCV that we like to term “internal recycling”, instead of recycling to the plasma membrane, as both compartments present similar membranal features.

Intracellular pathogens are generally thought to engage and subvert existing housekeeping endomembrane system pathways, such as the lysosomal or recycling pathway [38]. This pathogenic strategy is a hallmark of *Salmonella* invasion into epithelial cells, with many studies demonstrating complicated subversion patterns induced by *Salmonella* during invasion [4],[45]. In stark contrast, we report here a pathogenic strategy that does not exploit a pre-existing host pathway. Instead, *S*. *flexneri* induces the formation and trafficking of macropinosomes formed *in situ* in a “local pathogenic pathway” that involves the recruitment of Rab GTPases to macropinosomes, and does not involve recruitment of host endomembranes. Thus at each *S*. *flexneri* invasion site a local trafficking pathway is initiated without it being normally present in the cell. This pathway is subsequently exploited by the pathogen for vacuolar escape. The strategy employed by *S*. *flexneri* represents a divergence from existing paradigms of pathogen- host interactions centered on the successive pathogen subversion of established trafficking pathways [3]. In resemblance to other pathogenic systems, such as viral infection [46], macropinosomes are revealed to be a key constituent of the invasion process.

The establishment of a new model for *S*. *flexneri* invasion presented here hinged on the utilization of the emerging technique of C-FIB/SET. First, C-FIB/SET was used to demonstrate that *S*. *flexneri* enters cells within a tight BCV (Fig 2), likely explaining the lack of detectable fluorescent dextran labeling around bacteria when studied by light microscopy (Fig 1). This result strongly supports a model where bacterial entry occurs via a phagocytosis- like mechanism leading to tightly enveloped bacteria, and not, as is classically suggested (see introduction), via a macropinocytosis- like entry process—which would result in spacious, heterogeneous BCVs [2] (see Fig 6). In this “two parallel pathways” invasion model, the biological function of membrane ruffling and the resulting macropinosomes is related to the downstream step of vacuolar rupture, and not, as previously thought, to bacterial entry. Second, by specifically targeting rupturing vacuoles (as indicated by galectin-3-mOrange fluorescent labeling) for FIB/SET acquisition, this enigmatic and transient event became reliably and reproducibly accessible to 3D ultrastructural investigation for the first time, revealing macropinosome-BCV contacts and emanating dissociated membranes. The biological observations obtained through the application of C-FIB/SET in our study not only led to the emergence of new biological concepts regarding *S*. *flexneri* invasion, but act to demonstrate the power of this technique to investigate complex biological arenas containing multiple transient or rare events, complex 3D cellular organization and intricate structural interfaces.

We reveal a functional relationship between macropinosomes and the process of vacuolar rupture, mediated by the activity of Rab11. Macropinosome formation and trafficking ultimately result in macropinosome—BCV contact points formed at the onset of vacuolar rupture. How these contact points may drive membrane destabilization and eventual vacuolar rupture remains an open question. Our results lay the ground for future in depth investigations of the molecular mechanism and underlying biophysics driving this process, however initial insights into the mechanism of rupture can be gained from several observations: Firstly, in both fixed and dynamic light microscopy experiments, macropinosomes were never labeled with galectin-3-mOrange during vacuolar rupture. As BCV membranes become coated with galectin-3 at the loss of vacuolar integrity [36], fused or ruptured macropinosomes (i.e. with inner membranes exposed to the BCV or cytoplasm) would be expected to be labeled with galectin-3 as well. Furthermore, rupturing BCVs were never labeled with fluorescent dextran (fixed and pulse-chase experiments) or 2XFYVE-GFP (live microscopy; even during time-lapse studies with very high temporal resolution), as would be expected had dextran flowed from fusing macropinosomes into the collapsing BCV during rupture or if lipids were exchanged. These two observations argue against macropinosome -BCV fusion or macropinosome rupture as the underlying process facilitating BCV rupture, suggesting an altogether different mechanism is in place. Secondly, IpgD regulates macropinosome formation as well as rupture timing [25]. Infections with the Δ*ipgD* strain exhibit overall fewer macropinosomes in contact with rupturing BCVs, while the typical macropinosome-BCV contact point is still present in every rupturing BCV examined (Fig 4B). A decrease in the amount of contacts may limit damage to the BCV, explaining the observed delay in vacuolar rupture when using this strain and hinting at a cumulative damaging effect of macropinosome-BCV contacts. A decrease in macropinosome trafficking towards the BCV when using Rab11 KD or Rab11 DN would result in a similar effect. One possible mechanism could involve the formation of intraluminal vesicles (ILVs) pinched off from the BCV membrane and into maturing macropinosomes, causing increased membrane tension and eventually vacuolar rupture. Such vesicles are often observed at the interface between the BCV and connected macropinosome (Fig 5A, black arrowhead). The molecular machinery driving the formation of ILVs has been described in endosomes and phagosomes, but not in macropinosomes thus far to our knowledge [41]. Finally, the involvement of multiple bacterial effectors in vacuolar rupture (Fig 3A and 3B), without any single effector mutant inducing a complete arrest of rupture, implies that vacuolar rupture is not dependent on a single bacterial effector as described in other systems [47]. While other invasive bacterial pathogens employ direct lysis of their surrounding vacuole via bacterial proteins inserted directly into the membrane [48],[49], *S*. *flexneri* vacuolar rupture is most likely a complex process that involves multiple bacterial and host molecules acting to facilitate macropinosome formation, trafficking, contacts with the BCV and membrane destabilization. Future studies using imaging, biochemical and biophysical approaches will allow further elucidation of this critical step in the growth of *S*. *flexneri*.

Macropinosomes have been identified at the invasion site of intra-cellular bacterial pathogens years ago in seminal works by S. Falkow and others [11],[21],[50]. However, since then, their precise role in intra-cellular bacterial invasion has remained poorly understood and their importance overlooked. We report here a central role for macropinosomes in the pathogenic strategy of *S*. *flexneri*, and predict that this compartment is exploited by other invasive bacteria as well, possessing various biological functions in the context of pathogenicity yet to be discovered.

## Materials and Methods

### Bacterial strains

The following *S*. *flexneri* strains were used: M90T AfaI [22], expressing the adhesin afaI (Figs 1B, 2, 3C–3E and 4A–4C), the invasive Δ*ipgD* AfaI [30] (Figs 1B and 5B), M90T (Figs 1C and 4D), Δ*ipgD* (1C) and M90T expressing dsRed (Fig 1A and 1D). For the two mutant library screens (Fig 3A and 3B) all *S*. *flexneri* strains used were kindly provided by JR. Rohde (Dalhousie University): the strains do not express afaI and are a part of a pwR100 collection [51]. The parent strain for this collection is a streptomycin-resistant strain of *S*. *flexneri* serotype 5a (M90T-Sm). Growth medium was supplemented with ampicillin (50 μg/ml) for all strains except for Δ*ipgD* supplemented with tetracycline (5 μg/ml). All bacterial strains were cultured on tryptic casein soy broth (TCSB) agar plus 0.01% Congo red with 20mg/mL agar at 37°C.

### Cell culturing and infection conditions

Human epithelial HeLa cells (clone CCL-2 from ATCC) and NRK fibroblasts (Clone CRL- 1570 from ATCC) were cultured in Dulbecco’s modified Eagle’s medium (DMEM) supplemented with 10% (vol/vol) fetal bovine serum (FBS) at 37°C, 5% CO_2_. Caco-2 TC7 were kindly provided by P. Sansonetti (Institut Pasteur) and cultured as above except with 10% CO_2_. (Fig 1A, HeLa cells were used for all other experiments).

For invasion experiments, overnight bacterial cultures were inoculated at a 1/100 dilution in TCSB with the appropriate antibiotic if required and grown to an optical density of ≈ 0.4 at 600 nm (OD600). Prior to infection, bacteria were washed with PBS and resuspended in EM buffer (120 mM NaCl, 7 mM KCl, 1.8 mM CaCl_2_, 0.8 mM MgCl_2_, 5 mM glucose, 25 mM HEPES, pH 7.3) and incubated with poly-L-lysine for 15 min if they did not express AfaI. For imaging of fixed samples, bacteria were added to cells at MOI 30 (Figs 1A, 1C and 3A) or MOI 20 for all other fixed experiments and allowed to adhere for 10 minutes at RT. Samples where then incubated at 37°C for 30 minutes, washed three times in PBS and fixed with cold PFA 4% followed by staining with DAPI and Phalloidin. Dextran Alexa Fluor-488 and Alexa Fluor-647, 10,000 MW fixable (Life Technologies) were added to EM buffer and placed with cells for two to three hours before infection for sequential labeling assay (Fig 1D) or added during infection at a final concentration of 0.5μg/ml (all other experiments). For rupture timing screen (Fig 3B) and live imaging (Figs 3C–3E and 4A–4C) bacteria were added at a MOI of 50.

### Plasmids and transfection

HeLa cells were seeded into 96-well glass bottom plates (Greiner) at a density of 7000 cells per well 24 h prior to transfection. The cells were then transfected as described [25] with Rab11-GFP (Fig 4A–4C), 2XFYVE-GFP (Fig 3C–3E), galectin-3-mOrange (Figs 3C–3E, 4 and 5) or Rab11S25N-GFP, a Rab11 GDP-locked dominant negative (DN) (Fig 4D, kindly provided by B. Goud (Institut Curie)) using X-tremeGENE 9 reagent (Roche) for 24–48 h, according to the manufacturer’s instructions. For mutant library rupture timing screen (Fig 3B) cells were transfected with Actin-mOrange [30] and galectin-3-GFP [36] in an identical manner.

### Light microscopy

Fixed samples were imaged using a Perkin Elmer UltraView spinning disc confocal microscope, with a 60X/ 1.2NA water objective and a Z step size of 0.3 μm. For time lapse microscopy, cells were imaged with a confocal microscope (for experiments in Figs 3C–3E and 4A–4C) at 37°C with a 40X / 1.3 NA oil objective. Every 60, 30 or 5 seconds depending on experiment, a stack of 6 z-planes (step size of 0.5μm) was acquired sequentially in two or three channels using a 488nm, 561nm and 640nm laser depending on experiment. For mutant library rupture timing screen (Fig 3B) and Rab11 DN experiments (Fig 4D) cells were imaged at 37°C using a Nikon Ti-E wide-field microscope with a 20X air objective. Imaging was performed with excitation at 465 to 500 nm and 540 to 565 nm. Images were acquired every 90 or 120 seconds (depending on the exposure time) for 1 to 2 hours. In each screen experiment two mutants and one control (wild-type strain) were screened and images of 10 positions per strain acquired. For the Rab11 DN experiments 8 positions per condition were acquired with WT and IpgD as controls.

### Correlative large volume focused ion beam/ scanning electron tomography (C-FIB/SET)

Sample preparation and acquisition were performed as previously described [25]. In short, HeLa cells were cultured on MatTek dishes with finder grid (MatTek Corporations). Infections were performed as described above. Samples were fixed with 0.1% Glutaraldehyde and 4% paraformaldehyde for 30 minutes at room temperature. After high resolution confocal microscopy imaging (using 60X objective) positions of interest were marked using phase contrast and fluorescence with 10X and 20X objectives. Samples were then fixed overnight with 2.5% Glutaraldehyde in 0.1 M cacodylate buffer followed by fixation in 2.5% glutaraldehyde + 0.4% Tannic Acid pH 7.2 in 0.1 M cacodylate buffer for 30 min at RT. Samples were stained with 1% OsO4 in DDW for 30 min at 4°C. Samples were dehydrated in graded ethanol series and embedded in Epon followed by FIB/SET performed in a Helios Nanolab Dual beam (FEI) at the electron microscopy unit at the Weizmann Institute of Science (Israel). XY pixel size range was 6.5–8.3 nm and slice thickness 10 nm. Data was aligned using ImageJ (http://imagej.nih.gov/ij/). Amira and Avizo (FEI) were used for 3D visualization, data correlation, manual segmentation and supporting movies. Internalized bacteria were used as correlating fiducials. Inverted contrast is presented. Overall 15 C-FIB/SEM datasets of *S*. *flexneri* invasion sites were acquired, 11 of WT strain and 4 of Δ*ipgD* strain.

### Quantitative image analysis

Macropinosome size distribution (Fig 1A) was quantified using ICY (http://icy.bioimageanalysis.org/). “Spot detector” plugin was applied to segment dextran containing vesicles in 3D confocal stacks. A spherical assumption was used to extract vesicle diameter. 2300 vesicles from three separate experiments were analyzed. WT vs. Δ*ipgD* strain vesicles/bacterium (Fig 1C) was quantified using CellProfiler (http://www.cellprofiler.org/). Macropinosomes were counted only when found in actin labeled ruffles that contained at least one bacterium. Quantification is based on six separate experiments including in total 1186 cells, 1877 bacteria and 4330 vesicles. Unpaired t test was used for significance. Vesicle volume distribution (Fig 1D) was performed using a custom ICY protocol. 34 invasion sites in two experiments were chosen for analysis. *Shigella*-dsRed, dextran Alexa Fluor-488 and Alexa Fluor-647 signals were segmented in 3D. The sum of volumes of all vesicles of each type within 30 voxels of bacteria in each invasion site was calculated and normalized to the total vesicle volume at the site. Overall 476 vesicles were used in analysis. The mutant library macropinosome formation screen (Fig 3A) was quantified using CellProfiler in an identical manner to the WT vs. Δ*ipgD* strain vesicles formation assay described above. Results were obtained from two independent experiments, each containing at least 30 invasion sites, with 907 invasion sites analyzed in total. For mutant library rupture timing screen and Rab11 DN experiments (Figs 3B and 4D), time lapse series were visually analyzed with Fiji (http://fiji.sc). For rupture timing screen three independent experiments per strain were performed, at least 50 invasion events per strain were measured, with 791 events in total. For Rab11 DN experiments three independent experiments per condition were performed, with 946 rupture events analyzed in total (WT 351, IpgD 193, DN 402). Vacuolar rupture timing was measured as the time interval between the beginning of ruffle formation and the appearance of a Galectin-3 localized signal. Statistical analysis was performed in GraphPad Prism software v6. The difference between WT and mutants, or DN rupture timing was evaluated using one-way ANOVA. p < 0.05 was considered as significant: *p<0.05, **p<0.01, ***p<0.0001, and ****p<0.0001. Time lapse microscopy (Fig 3C–3E) was manually quantified using Fiji, with bordering event counted when 2XFYVE-GFP and galectin-3-mOrange signal where found within 6 pixels distance of each other. Analysis of macropinosomes bordering at the onset of BCV rupture was performed by manually counting all instances of 2XFYVE-GFP positive vesicles present at the rupture site at least one frame before galectin-3 cage appearance (positive hit) in contrast to vesicles appearing only during or after galectin-3 cage appearance (negative hit). 36 events were imaged in eight independent experiments All quantitative data in the manuscript is presented as mean, with error bars presented as s.d.

Supporting figures materials and methods can be found in S1 Text.

## Supporting Information

S1 Text Supporting information materials and methods. (DOCX)

S1 Fig Complementation experiment for Δ*ipgD* strain. Invasion sites formed after a 30 min infection were analyzed. Number of vesicles per invasion site was calculated using automated software (n = 105 invasion sites analyzed in three independent experiments). Δ*ipgD*/IpgD shows partial complementation. Unpaired student t test was used for significance.
(TIF)

S2 Fig C-FIB/SET confirms the presence of newly formed vesicles at the invasion site. C-FIB/SET was applied to correlate dextran Alexa Fluor-647 labeling (purple) added during 30 min infection to all vesicles detected by FIB/SET at the invasion site (EM Segmentation, orange). Bacteria are labeled by DAPI (cyan) and segmented from FIB/SET data (blue). Two separate datasets are presented, (A) and (B). Quantitative analysis reveals that 95% and 99% (respectively) of vesicles found at the invasion site reside within the fluorescent dextran label. Vesicles outside of dextran label are highlighted in the overlay view (yellow). In data set B, five of the largest vesicles that reside outside of the dextran labeling are found near the surface of the cell, most likely representing late forming macropinosomes formed after removal of dextran in the washing phase. Scale bars are 10μm.
(PNG)

S3 Fig Full mutant screens results, application of the Arp2/3 inhibitor CK-666 and high temporal resolution imaging of vacuolar rupture. (A) Full results of the two bacterial effector mutant library screens described in Fig 3A and 3B. All bacterial effectors examined by both screens are presented. Results are normalized to WT. (B) CK-666 inhibits macropinosome formation (left) and causes a significant delay in vacuolar rupture (middle), but does not inhibit bacterial entry (right). Unpaired student t-test is used for significance. See supplementary materials and methods for experimental details. (C) 2XFYVE-GFP is partially co-localized with dextran positive vesicles at the invasion site. Cells transfected with 2XFYVE-GFP were infected for 30 min with WT strain in the presence of dextran Alexa Fluor-647. 2XFYVE-GFP was partially co-localized with dextran positive vesicles at the invasion site. (D) High temporal time lapse microscopy reveals macropinosomes border the BCV at the onset of vacuolar rupture. Cells transfected with the PI3P marker 2XFYVE-GFP, and the vacuolar rupture marker galectin-3-mOrange, were imaged in three dimensions (z-interval: 0.5 μm,) during *S*. *flexneri* invasion at five second intervals. Z-projections are shown in the figure. After the onset of membrane ruffling (0s), a transient PI3P enrichment was observed (130s) around the BCV with a lifetime of 20-250s (n = 27), followed by macropinosome formation (310s). Vacuolar rupture begins with macropinosomes bordering the BCV (340s) in 33 out of 36 events observed (92%). Macropinosomes persist around the BCV during rupture (440s). Scale bar is 5μm. See also S4 Movie. For PI3P cage lifetime analysis, 27 events from seven independent experiments were analyzed.
(PNG)

S4 Fig Rab GTPases recruitment to dextran labeled macropinosomes at the invasion site. (A) Cells transfected with Rab5-GFP, Rab7-GFP or Rab11-GFP were infected with *S*. *flexneri* expressing dsRed for 30 minutes in the presence of dextran Alexa Fluor 647 for 30 minutes, followed by washes and fixation. (B) Cell transfected with Rab11S25N-GFP, a GDP locked Rab11 dominant negative, and infected as in (A). Representative images are shown, scale bars are 10 μm. (C) Rab11 WT vs. Rab11S25N-GFP ruffling formation.
(PNG)

S1 Movie Connected to Fig 2A: C-FIB/SET provides detailed 3D structural information of discreet and transient stages of *S*. *flexneri* invasion. 3D confocal stack of an invasion site in a cell infected with *S*. *flexneri* and labeled with DAPI (cyan) and actin-phalloidin (Magenta) is presented followed by a view of the FIB/SET data from three different axes. Finally a segmentation showing membrane ruffles is presented.
(MPG)

S2 Movie Connected to Fig 2B: The BCV is a tight compartment distinct from surrounding vesicles. The detailed structure of the BCV is presented. Presentation of the ultrastructural data is followed by a segmentation of the bacterial cytosol and membrane (blue), LPS layer (white), BCV membrane (yellow), actin cage (magenta) and surrounding vesicles (orange). A cut-out-view in the context of membrane ruffling is presented.
(MPG)

S3 Movie Connected to Fig 3C: Time lapse microscopy reveals macropinosomes border the BCVs during vacuolar rupture. Confocal time lapse microscopy of wild-type strain infection of cells transfected with the macropinosome marker 2XFYVE-GFP and vacuolar rupture marker galectin-3-mOrange. Images were taken every 30 s, z-projections are presented, scale bar is 5μm. Channels are from left to right- 2XFYVE-GFP dextran, galectin-3-mOrange, composite.
(AVI)

S4 Movie Connected to S3D Fig: High temporal resolution time lapse microscopy reveals macropinosomes border the BCV at the onset of vacuolar rupture. Cells transfected with the PI3P marker 2XFYVE-GFP, and the vacuolar rupture marker galectin-3-mOrange, were imaged with confocal microscopy (z-interval: 500 nm,) during S. flexneri invasion at five second intervals. Images were taken every 30 s. Z-projections are shown in the movie, scale bar is 5 μm. Channels are from left to right- 2XFYVE-GFP dextran, galectin-3-mOrange, composite
(AVI)

S5 Movie Connected to Fig 4A–4C: Rab11 is directly and dynamically recruited to macropinosomes. Dextran pulse chase live imaging experiment. Cells transfected with Rab11-GFP and galectin-3-mOrange were infected with *S*. *flexneri* in the presence of dextran Alexa Fluor 647. After 22 minutes dextran was washed out and live imaging was initiated. Channels are from left to right- Rab11-GFP, dextran Alexa Fluor 647, galectin-3-mOrange, composite. Scale bar is 5 μm.
(AVI)

S6 Movie Connected to Fig 4D: Rab11 activity is required for efficient vacuolar. Representative movie showing live imaging experiment of cells transfected with Rab11-GFP and gal-3mOrange, and infected with *S*. *flexneri*. Channels are from left to right- Rab11-GFP, galectin-3-mOrange, composite. Scale bar is 10 μm
(AVI)

S7 Movie Connected to Fig 4D: Rab11 activity is required for efficient vacuolar rupture. Representative movie showing live imaging experiment of cells transfected with Rab11S25N-GFP dominant negative (DN) and gal-3mOrange, and infected with *S*. *flexneri*. Channels are from left to right- Rab11S25N-GFP, galectin-3-mOrange, composite. Scale bar is 10 μm.
(AVI)

S8 Movie Connected to Fig 5A: The bacteria containing vacuole and macropinosomes come into direct contact during vacuolar rupture. Wild-type strain. 3D confocal view of an invasion site in a cell transfected with galectin-3-mOrange rupture marker (red) and labeled with DAPI (cyan) is presented. The FIB/SET data set is shown followed by segmentation of a bacterial cytosol and LPS (blue, white), dissociated BCV membrane (yellow) and macropinosomes in direct contact with the BCV membrane (orange). Frame showing dissociated membrane emanating from the macropinosome-BCV contact point is highlighted.
(MPG)

S9 Movie Connected to Fig 5B: The bacteria containing vacuole and macropinosomes come into direct contact during vacuolar rupture. Δ*ipgD* mutant strain. Movie notation and progression identical to S8 Movie.
(MPG)
