# Supplementary material for: Macropinosomes are Key Players in Early Shigella Invasion and Vacuolar Escape in Epithelial Cells
Source: PLoS Pathog. 2016 May 16;12(5):e1005602. doi: 10.1371/journal.ppat.1005602 (PMC4868309; doi:10.1371/journal.ppat.1005602)
Supplement: S1 Text — (DOCX) [file ppat.1005602.s001.docx]

**S1 Text. Supporting information materials and methods**

The following strains were used: M90T Afai (S1 Fig, S2 Fig, S3B-D Fig) and Δ*ipgD* Afai expressing the adhesin afaI, Δ*ipgD*/IpgD Afai complementation protein in trans (S1 Fig) and M90T expressing dsRed (S4A, S4B Fig). Strains used in the mutant screens (S3A Fig) were kindly provided by JR. Rohde (Dalhousie University) and described in the main text. Cell culture and transfections were performed as described in the main text with HeLa cells used for all experiments. Infections were performed as described in the main text with MOI of 30 (S1 Fig, S3B Fig, S4 Fig), 20 (S2 Fig, S3C Fig) or 50 (S3D Fig). Light microscopy and C-FIB/SET were performed as described in the main text. Quantitative image analysis of complementation mutants (S1 Fig) was performed using CellProfiler (<http://www.cellprofiler.org/>) with 105 invasion sites analyzed in three independent experiments. Unpaired student t test was used to determine significance. Error bars are in s.d.. Amira and Avizo (FEI) were used for data segmentation, correlation and vesicle counting (S2 Fig). Mutant screens experimental procedures and data analysis are as described in the main text (S3A Fig). For macropinosome formation inhibition by CK-666 (Sigma) (S3B Fig) cells were infected with WT strain for 30 minutes in the presence of fluorescent of dextran and with 1mM CK-666 or untreated controls. Cell were washed, fixed and stained with DAPI and phalloidin. Macropinosome number per foci were quantified using CellProfiler software. Overall 142 invasion sites containing 1679 vesicles were counted (73 invasion sites, 1162 vesicles for control, 69 invasion sites, 517 vesicles for drug) in three independent experiments. Only invasion sites containing at least one vesicle were used for analysis. Unpaired student t test was used to determine significance. Error bars are in s.d. For CK-666 inhibition of vacuolar rupture (S3B Fig), movies of WT infections of treated and untreated cells (performed as described in the main text for figure 3B) were visually analyzed with Fiji (http://fiji.sc). Four (CK-666) independent experiments per condition were performed, with 830 rupture events analyzed in total (Control 489, Drug 341). Vacuolar rupture timing was measured as the time interval between the beginning of foci formation and the appearance of a Galectin-3 localized signal. For Rab11 DN ruffling formation (S4C Fig) three independent experiments per condition were performed, with 946 events analyzed in total (WT 351, DN 402). Statistical analysis was performed in GraphPad Prism software v6. The difference between conditions was evaluated using one-way ANOVA. p < 0.05 was considered as significant: *p<0.05, **p<0.01, ***p<0.0001, and ****p<0.0001. All quantitative data in the manuscript is presented as mean, with error bars presented as s.d.
