# Supplementary figures and images for: Macropinosomes are Key Players in Early Shigella Invasion and Vacuolar Escape in Epithelial Cells
Source: PLoS Pathog. 2016 May 16;12(5):e1005602. doi: 10.1371/journal.ppat.1005602 (PMC4868309; doi:10.1371/journal.ppat.1005602)

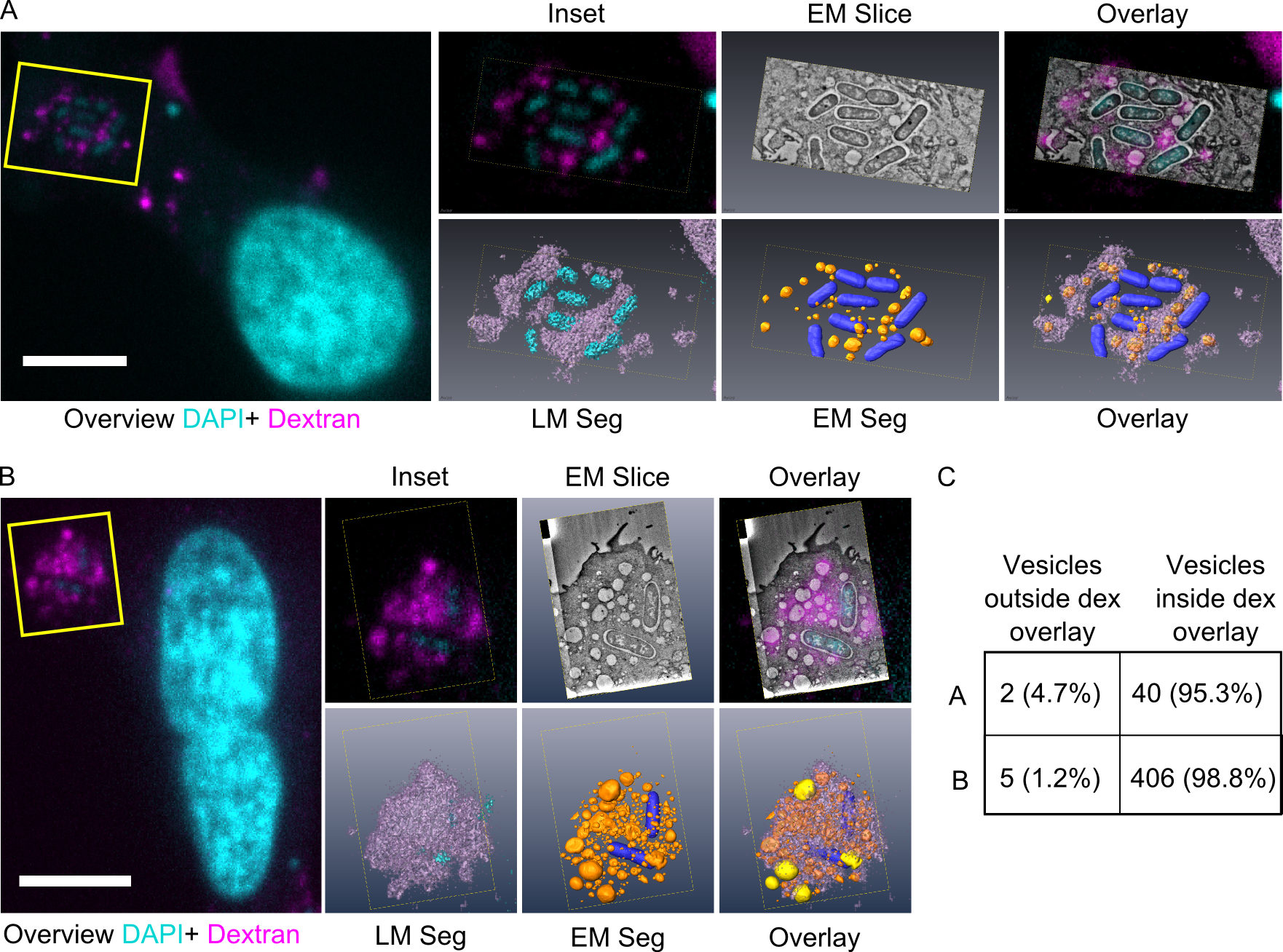

Supplement: S2 Fig — C-FIB/SET was applied to correlate dextran Alexa Fluor-647 labeling (purple) added during 30 min infection to all vesicles detected by FIB/SET at the invasion site (EM Segmentation, orange). Bacteria are labeled by DAPI (cyan) and segmented from FIB/SET data (blue). Two separate datasets are presented, (A) and (B). Quantitative analysis reveals that 95% and 99% (respectively) of vesicles found at the invasion site reside within the fluorescent dextran label. Vesicles outside of dextran label are highlighted in the overlay view (yellow). In data set B, five of the largest vesicles that reside outside of the dextran labeling are found near the surface of the cell, most likely representing late forming macropinosomes formed after removal of dextran in the washing phase. Scale bars are 10μm. (PNG) [file ppat.1005602.s003.png]

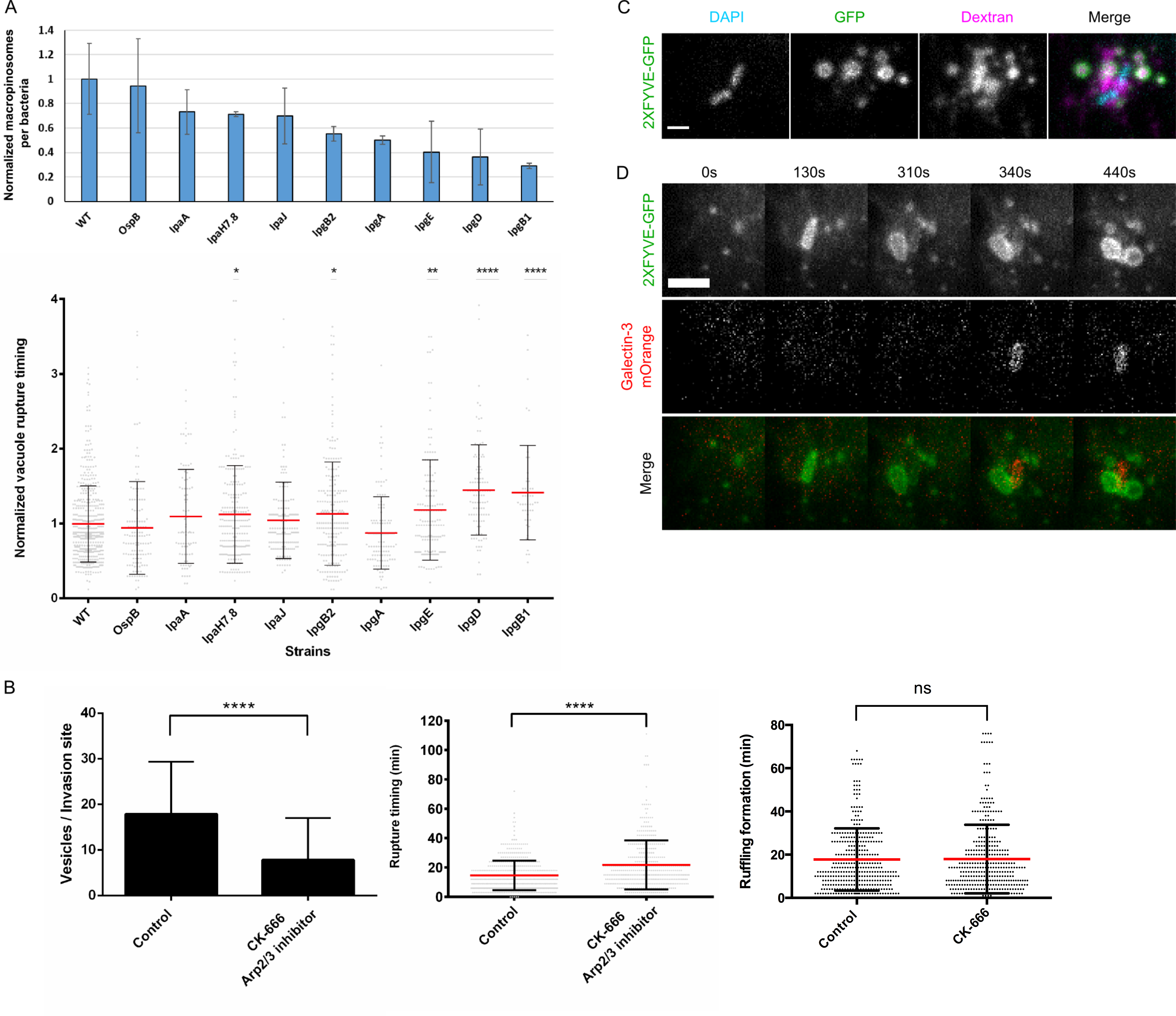

Supplement: S3 Fig — (A) Full results of the two bacterial effector mutant library screens described in Fig 3A and 3B. All bacterial effectors examined by both screens are presented. Results are normalized to WT. (B) CK-666 inhibits macropinosome formation (left) and causes a significant delay in vacuolar rupture (middle), but does not inhibit bacterial entry (right). Unpaired student t-test is used for significance. See supplementary materials and methods for experimental details. (C) 2XFYVE-GFP is partially co-localized with dextran positive vesicles at the invasion site. Cells transfected with 2XFYVE-GFP were infected for 30 min with WT strain in the presence of dextran Alexa Fluor-647. 2XFYVE-GFP was partially co-localized with dextran positive vesicles at the invasion site. (D) High temporal time lapse microscopy reveals macropinosomes border the BCV at the onset of vacuolar rupture. Cells transfected with the PI3P marker 2XFYVE-GFP, and the vacuolar rupture marker galectin-3-mOrange, were imaged in three dimensions (z-interval: 0.5 μm,) during S. flexneri invasion at five second intervals. Z-projections are shown in the figure. After the onset of membrane ruffling (0s), a transient PI3P enrichment was observed (130s) around the BCV with a lifetime of 20-250s (n = 27), followed by macropinosome formation (310s). Vacuolar rupture begins with macropinosomes bordering the BCV (340s) in 33 out of 36 events observed (92%). Macropinosomes persist around the BCV during rupture (440s). Scale bar is 5μm. See also S4 Movie. For PI3P cage lifetime analysis, 27 events from seven independent experiments were analyzed. (PNG) [file ppat.1005602.s004.png]

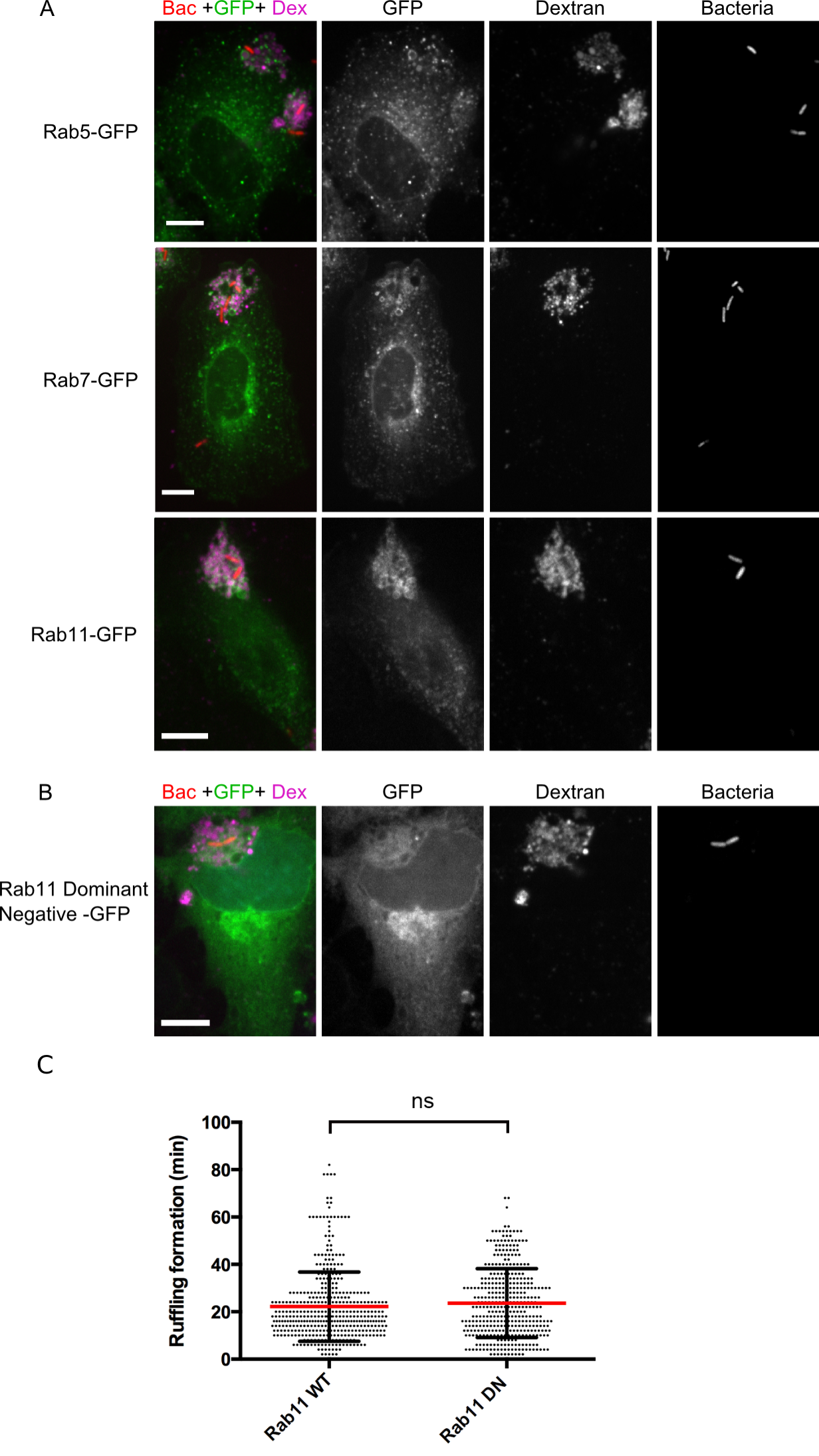

Supplement: S4 Fig — (A) Cells transfected with Rab5-GFP, Rab7-GFP or Rab11-GFP were infected with S. flexneri expressing dsRed for 30 minutes in the presence of dextran Alexa Fluor 647 for 30 minutes, followed by washes and fixation. (B) Cell transfected with Rab11S25N-GFP, a GDP locked Rab11 dominant negative, and infected as in (A). Representative images are shown, scale bars are 10 μm. (C) Rab11 WT vs. Rab11S25N-GFP ruffling formation. (PNG) [file ppat.1005602.s005.png]
